# DNA replication stress as an Achilles' heel of cancer

**DOI:** 10.18632/oncotarget.3147

**Published:** 2014-12-30

**Authors:** Caterina Da-Rè, Thanos D. Halazonetis

**Affiliations:** Department of Molecular Biology, University of Geneva, Geneva, Switzerland

There is now significant evidence indicating that replication stress (RS) is prevalent in human cancers and that it is induced by the constitutively activated oncogenes that are driving cancer cell proliferation [1]. The collapsed replication forks and DNA damage associated with RS are most likely a significant driver of the genomic instability present in human cancers. At the same time, DNA damage activates the p53 tumor suppressor protein, which induces senescence or apoptosis. Thus, RS provides a selective advantage to inactivate *p53*, thereby explaining the high frequency of *p53* mutations in human cancers. Supporting this sequence of events, RS is evident in precancerous lesions before overt genomic rearrangements and *p53* mutations [1].

RS is also likely to affect the response of cancer cells to therapy. Indeed, most of the empirically identified cancer therapies that are used today as first-line therapy have some connection to RS. For example, gemcitabine and cytarabine are deoxycytidine analogs; camptothecin, etoposide and adriamycin (doxorubicin) are topoisomerase inhibitors; and alkylating agents, cisplatin and irradiation induce DNA lesions, which stop or impede fork progression. The efficacy of these agents could reflect the high proliferation rate of cancer cells. However, the presence of RS in cancers could also be important, as cancer cells already have “difficulty” replicating their genomes and are, therefore, more sensitive to the treatment than normal cells.

A recent study supports the above argument. The degree of RS in a cohort of diffuse large B-cell lymphomas (DLBCLs) correlated to survival of patients treated with standard therapies [2]. This correlation held even when patients with similar international prognostic indices were compared. A previous study examining patients with early operable non-small cell lung cancer reported similar findings [3].

RS may not only serve as a prognostic indicator, but, perhaps could also be exploited for development of more effective therapies. Both checkpoint and repair pathways are activated in response to RS. The RS checkpoint is mediated by the kinase ATR, which phosphorylates and activates the kinase Chk1; in turn, Chk1 inhibits cell cycle progression. ATR and Chk1 inhibitors are at various stages of preclinical and clinical testing. ATR inhibitors potentiate RS and exhibit synthetic lethality with cancer-associated mutations in tissue culture cell systems. In the clinic, phase I studies are underway to assess their safety in patients with late-stage cancers. While no results regarding efficacy are yet available from the clinical studies, hypomorphic *ATR* alleles in mouse models prevent the development of *Myc*-driven B-cell lymphomas [4].

Chk1 inhibitors, which are at a more advanced stage of development than ATR inhibitors, have been tested in the clinic either as single therapies or in combination with deoxycytidine analogs [5]. Preliminary evidence for anti-tumor activity has been reported by one study. However, cardiotoxicity has also been reported, suggesting that further development of these agents may be needed. In a mouse model of B-cell lymphoma, a Chk1 inhibitor induced strong regression of the tumors via apoptosis, but only minimally affected the normal tissues [4]. Similarly, a highly selective Chk1 inhibitor induced apoptosis of primary cells derived from human DLBCLs and other lymphomas with high levels of RS, whereas normal bone marrow cells were unaffected [2]. Thus, these and other studies suggest that inhibitors of RS checkpoint proteins can target preferentially cancer cells over normal cells.

The pathways that repair stalled and collapsed DNA replication forks could also be therapeutic targets. Break-induced replication (BIR) repairs collapsed forks in cells with oncogene-induced RS [6]. Inhibiting BIR arrest proliferation of cancer cells, but has little effect on normal cells. Pathways involved in preserving the integrity of stalled replication forks are also likely to be potential targets for novel cancer therapies [7].

The last few decades have seen an exponential increase in our understanding of cancer at the molecular level. This knowledge has already led to the development of novel therapeutic agents, including inhibitors of oncogenes. While these inhibitors have had a major impact on disease-free progression in some cancers, in many cases the benefits are not long-lasting. Given the ease with which cancer cells develop resistance to therapy, it is now thought that targeting several pathways simultaneously may be the most promising strategy. Along these lines, agents that modulate the response of cells to RS could provide important benefits to cancer patients. The future will tell.
